# The Hierarchy of Protoxylem Groupings in Primary Root and Their Plasticity to Nitrogen Addition in Three Tree Species

**DOI:** 10.3389/fpls.2022.903318

**Published:** 2022-06-23

**Authors:** Zhongyue Li, Siyuan Wang, Wenna Wang, Jiacun Gu, Yan Wang

**Affiliations:** ^1^Mountain Tai Forest Ecosystem Research Station of State Forestry and Grassland Administration, College of Forestry, Shandong Agricultural University, Tai’an, China; ^2^Key Laboratory of Sustainable Forest Ecosystem Management-Ministry of Education, School of Forestry, Northeast Forestry University, Harbin, China; ^3^Institute of Tropical Agriculture and Forestry, Hainan University, Haikou, China

**Keywords:** hardwood species, fine root, root developmental order, protoxylem group, morphology, anatomy, fertilization

## Abstract

Protoxylem grouping (PG), a classification based on the number of protoxylem poles, is a crucial indicator related to other functional traits in fine roots, affecting growth and survival of individual root. However, within root system, less is known about the arrangement of PG. Moreover, the responses of PG to fertilization are still unclear. Here, we selected three common hardwood species in Northeast China, *Juglans mandshurica*, *Fraxinus mandshurica,* and *Phellodendron amurense*, conducted root pruning and nutrient addition. In this study, we analyzed the PG, morphology, and other anatomy traits of newly formed root branches. The results showed all root length, diameter, and stele, as well as hydraulic conductivity, were significantly positive related to the PG number, and the PG number generally decreased with ascending root developmental order; these patterns were independent of species and fertilization. Additionally, we also found the plasticity of PGs to environmental changes, in terms of the increased frequency of high PG roots after fertilization, significantly in *J. mandshurica* and *F. mandshurica*. Therefore, the heterogeneity, hierarchy, and plasticity of individual roots within root system may be widespread in woody plants, which is of great significance to deepen our understanding in root growth and development, as well as the belowground ecological process.

## Introduction

**Figure 1:**
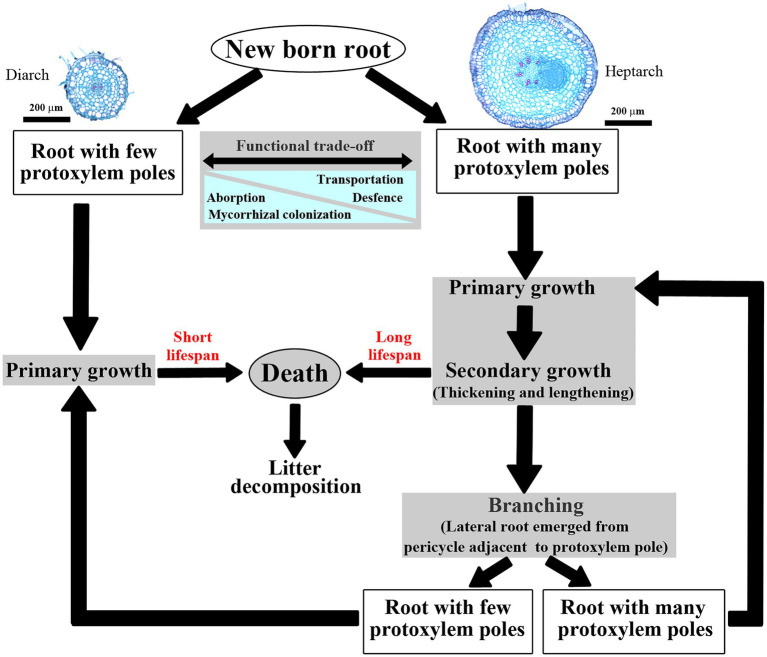
Life cycles of an individual newly formed root. Illustrated two typical roots of *Fraxinus mandshurica* have two (diarch) and seven (heptarch) protoxylem groups, respectively.

Fine root anatomical traits are crucially fundamental for root branching architecture and resource absorption (Ma et al., 2018; Lynch, 2019, 2021; Freschet et al., 2021a,b; Valverde-Barrantes et al., 2021; Galindo-Castañeda et al., 2022). In primary roots, stele (vascular cylinder) is responsible for resource axial transportation (Gu et al., 2014; Kong et al., 2014), which is tightly associated with the number and diameter of conduits (Wang et al., 2018, 2019). Individual roots have one or more protoxylem group (PG, i.e., protoxylem pole), correspondingly, which could be classified into monarch, diarch, triarch, tetrarch and so on depending on the number of PG. Furthermore, PG is an important indicator linked to the architecture and function of individual roots (Baba et al., 2018, 2019) and even the whole root system (Hishi, 2007; Tawa and Takeda, 2015; Figure 1).

For example, PG strongly influences the potential trade-offs of absorption and transportation (Zadworny and Eissenstat, 2011; Bagniewska-Zadworna et al., 2012, 2014). In high PG (PG ≥ 4 or 5) roots, thicker stele and wider conduit would increase axial transport efficiency (Bagniewska-Zadworna et al., 2012, 2014). However, this transportation ability of high PG roots was enhanced at the expense of absorptive capacity because of a complete lack of mycorrhizal colonization (Zadworny and Eissenstat, 2011). Similarly, low PG (PG ≤ 3 or 2) roots were relatively stronger in absorption but weaker in transportation because of their greater intensity of mycorrhizal colonization and less developed xylem elements (Zadworny and Eissenstat, 2011; Bagniewska-Zadworna et al., 2012, 2014). And, PG also tightly associates with root lifespan (Hishi and Takeda, 2005a). Primary roots with high PG would advance to secondary growth (Hishi and Takeda, 2005a; Hishi, 2007) owing to the activity of vascular cambium, manifested as the protoxylem expansion, cortical parenchyma cells shrinkage and strong suberin deposition (Gambetta et al., 2013; Tawa and Takeda, 2015). However, these high PG roots with secondary growth were more readily defending against biotic and abiotic challenges, converting into perennial ones (Hishi and Takeda, 2005a,b), whereas the low PG roots usually died as primary roots before becoming secondary growth (Hishi, 2007). Additionally, PG closely links to root branching architecture. Generally, lateral roots emerge from pericycle cells adjacent to the protoxylem poles (Ötvös and Benková, 2015), thus primary roots with high PG would be more likely to branch (McMichael et al., 1987; Chen et al., 2018). For instance, if the primary root of *Hakea prostrata* was triarch, then thousands of densely rootlets would regularly space along parental root axis in three longitudinal rows (Shane and Lambers, 2005; Lambers and Shane, 2007). Similar and even more complex root branching architectures also occurred in other parent roots containing four (*Arachis hypogaea*, Yarbrough, 1949), five (*Allium cepa*, Pulgarin et al., 1988) or six (*Quercus rubra*, Lyford, 1980) PGs. Therefore, PG as an indicator of individual roots traits would supply a new viewpoint to classify heterogeneous roots. However, the significance of PG has only been demonstrated in few tree species (Zadworny and Eissenstat, 2011; Tawa and Takeda, 2015).

Previous researches had revealed that the PG arrangement showed large inter- and intra-specific variations. Firstly, according to the results reported in botany and fossil studies, the number of PG was generally highest in monocots (Sargant, 1903; Holm, 1904), and followed by dicots (Compton, 1912, 1913; Whitaker, 1923; Wardlaw, 1928), gymnosperms (Worsdell, 1906; Dorety, 1908) and ferns (Barry, 1884; Wardlaw, 1928) species. Secondly, the number of PG was highly associated with branching position, newly formed framework original roots had more PGs than lateral ones among woody species, i.e., four or five PGs vs. two PGs (Eissenstat and Achor, 1999; Zadworny and Eissenstat, 2011; Bagniewska-Zadworna et al., 2012). Additionally, PG arrangement was also affected by some external factors. For instance, roots colonized by mycorrhizal (*Allium porrum*, Fusconi et al., 1994) and pathogenic fungi (*Nicotiana glauca*, Esau, 1941), or treated by auxin (*Pisum sativum*, Torrey, 1957; *Vaccinium virgatum*, Baba et al., 2018) and manure (*Hordeum vulgare*, Jackson, 1922) would have more PGs than the control ones. However, most previous PG studies have been focused on herbaceous species. In woody plants, how PG arrangement occurs within root system, and how PG composition responds to changed soil nutrient availability after soil disturbance, which remain open issues that require further investigation.

Furthermore, the PG number in root cross sections that does not change throughout the life cycle of individual roots (Wilson and Horsley, 1970; Hishi and Takeda, 2005b), therefore, it is crucial to adopt optimal root dissection approach to differentiate the roots emerged from different positions. In last decades, the Strahler’s stream ordering system was widely used for root form and function analysis (Pregitzer et al., 2002; Guo et al., 2008a; McCormack et al., 2015). According to the root stream order, a simple unbranched root is first-order, after the emergence of laterals, then part of the original first-order root would become second- or higher-order (Fitter, 1982), even though these different order root segments have same PG arrangement. Therefore, if the root stream order was applied in the PG analysis, which would cause a repetition or overestimation of PG quantity statistics. Alternatively, root developmental order was efficient to describe the PG arrangement across different root architectures (Baba et al., 2018), because it corresponds with the ontogeny of root system (Pregitzer et al., 1997). Specifically, the first-order roots are the original roots, these are the mother/parent roots of finely branched second-order roots, and third-order roots are the daughter roots of the second-order ones (Picon-Cochard et al., 2012). Thus, all different order roots had apical meristem with primary growth, and first-order roots are thicker in diameter than other higher-order roots (Picon-Cochard et al., 2012). In this classification, most first-order roots were categorized as pioneer roots (=framework or skeletal roots), and high-order roots were fibrous roots (=short or feeder roots; Zadworny and Eissenstat, 2011; Bagniewska-Zadworna et al., 2012), which were primarily responsible for the resource transportation and absorption, respectively.

Here, three hardwood species, *Juglans mandshurica*, *Fraxinus mandshurica* and *Phellodendron amurense*, were chosen in Northeast China, which were markedly different in root branching architecture and root functional traits (Wang et al., 2016, 2020). After root pruning and following fertilization performed in the field, newly formed root branches were harvested, the functional traits of different root developmental order were also compared. The first aim of this study was to explore the correlations between PG number and other root functional traits. As the tight associations of PG number and root functional traits is clarified in Figure 1, we hypothesized that the number of PG was positively related to root length, root diameter, stele diameter and hydraulic conductivity. Next, we aimed to investigate the PG arrangement along root developmental order. According to the viewpoint of classical plant anatomy, laterals that emerged from the primary root axis often show a reduced PG number in herbaceous (*P. sativum*, Torrey, 1955). Therefore, our second hypothesis was that the number of PG would decrease with the increase of root developmental order in woody species. Meanwhile, we also tried to verify the effect of fertilization on the PG composition in woody species. A previous study had reported the application of superphosphate and nitrate or superphosphate and potash would induce more PGs in barley roots (Jackson, 1922). In such a manner, our third hypothesis was that the proportion of high PG in newly formed roots would increase after nitrogen addition across woody species.

## Materials and Methods

### Study Site

The research was conducted at the Maoershan Forest Research Station of Northeast Forestry University (45°21′-45°25′ N, 127°30′-127°34′ E, with a mean elevation of 300 m), in Heilongjiang Province, China. The site has a temperate continental monsoon climate with an average annual temperature of 2.8°C, lowest temperature in January and highest temperature in July of −19.6°C and 20.9°C, respectively. The annual precipitation ranges from 600 to 800 mm, of which 80% falls in June, July, and August, and the growing season is about 120–140 days (Wang et al., 2006). Soil is Hap-Boric Luvisols with well-developed horizons and are well drained (Gong et al., 1999). Plantations of three species were established in 1986 by planting nursery-raised 2-year-old bare root seedlings using a 1.5 m × 2.0 m planting grid. Mean standing density was 3,003, 3,187 and 2,884 tree ha^−1^ for *J. mandshurica*, *F. mandshurica* and *P. amurense*, respectively.

### Root Pruning, Nutrient Addition and Harvesting

In early May 2015, root pruning approach was used in plantations of three species (Eissenstat et al., 2015; Liu et al., 2015). In each plantation, a 20 × 20 × 10 cm^3^ soil block was selected randomly. For each block, woody roots were traced back to an identified tree, roots from shrubs or herbaceous were removed based on root morphology, color and elasticity. Then *c.* 2-5-mm-diameter and *c.* 10-cm-length woody roots were selected, and the distal end of the woody roots and the lateral fine roots were trimmed using scissors. Then, the selected woody roots were reburied with original but sieved fresh soil, and covered with original litters, and watered. To ensure the sampling size of harvested root branches would meet the statistical analysis, for each species, we chose 7 trees and sampled 14 soil blocks (2 blocks per tree). 5–6 woody roots per soil block were selected. Totally, 70 woody roots were selected. For two soil blocks of individual trees, one was assigned randomly for the nutrient addition treatments (NH_4_NO_3_, at a rate of 10 g Nm^−2^a^−1^), and the other was control. In early July, the second nutrient addition was conducted. Specifically, the fertilizer was broadcasted evenly within the chosen soil block, without dissolved in water.

After incubation time of 118 days, newly growing root branches were collected carefully by cutting the woody roots in middle September 2015. All the root clusters were immediately placed in a cooler and transported to the laboratory. In the laboratory, the intact root samples were washed with deionized water gently to remove the soil adhered to roots, then kept in formalin-aceto-alcohol (FAA) solution (90 ml 50% ethanol +5 ml 100% glacial acetic acid +5 ml 37% methanol), and stored in a 4°C refrigerator for anatomical and morphological analysis.

### Anatomical and Morphological Analysis

**Figure 2:**
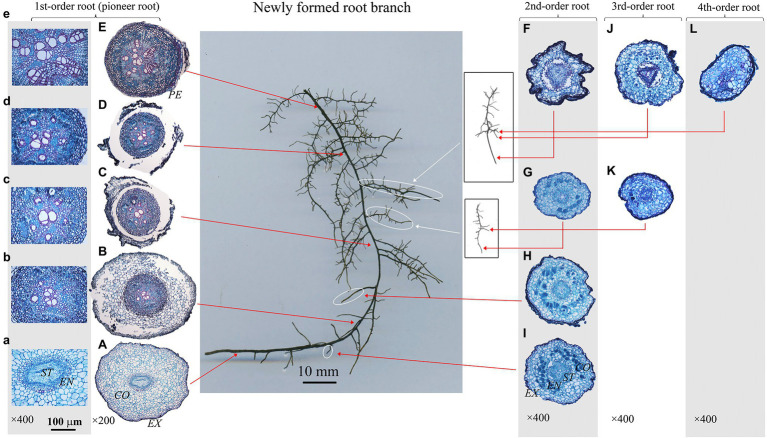
Protoxylem group (PG) arrangement along root developmental order of *Juglans mandshurica* under control. There were five PGs **(A,B)** in first-order root, four PGs **(F–I)** in second-order roots, three **(J)** and two **(K)** PGs in third-order roots, and two **(L)** PGs in fourth-order root, respectively. The nature of PG in first-order root was obvious in primary growth **(A,B)**, but lost in the secondary growth **(C–E)**. *EX*, exodermis; *CO*, cortex; EN, endodermis; *ST*, stele; *PE*, periderm.

**Figure 3:**
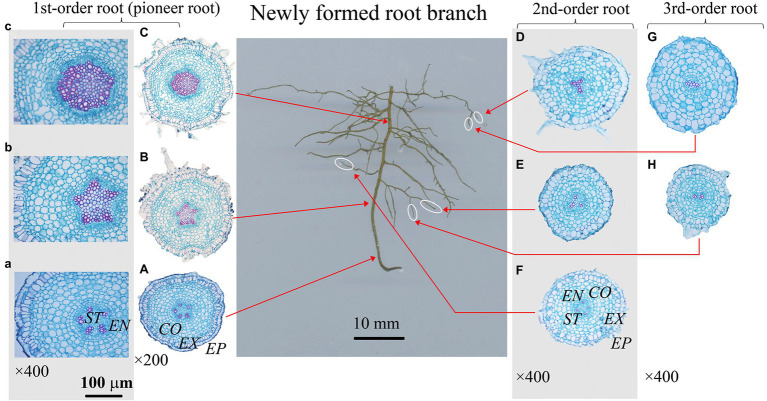
Protoxylem group (PG) arrangement along root developmental order of *Fraxinus mandshurica* under control. There were five protoxylem groups (PGs; **A,B**) in first-order root, four **(F)** and three **(D,E)** PGs in second-order roots, and two PGs **(G,H)** in third-order roots. The nature of PG in first-order root was obvious in primary growth **(A,B)**, but lost in the secondary growth **(C)**. *EP*, epidermis; *EX*, exodermis; *CO*, cortex; EN, endodermis; *ST*, stele.

**Figure 4:**
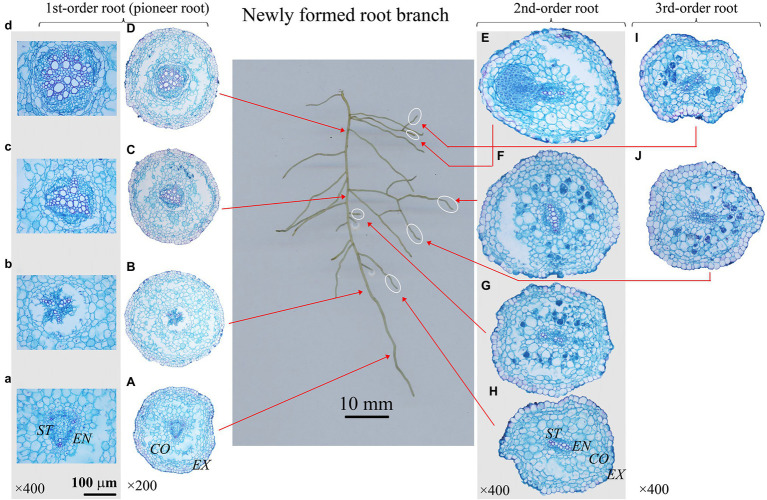
Protoxylem group (PG) arrangement along root developmental order of *Phellodendron amurense* under control. There were three PGs **(A-C)** in first-order root, and two PGs **(E-J)** in second- and third-order roots. The nature of PG in first-order root was obvious in primary **(A-C)**, but lost in secondary growth **(D)**. *EX*, exodermis; *CO*, cortex; EN, endodermis; *ST*, stele.

In this study, root dissections were conducted based on the classic root developmental approach (Fitter, 1982; Pregitzer et al., 1997), a root branch was classified beginning with the primary (first-order or pioneer) root and increasing sequential with each branch from proximal to distal portion of the root system (Figures 2–4). Here, we used this developmental approach/order because it corresponds with the ontogeny of the root system (Pregitzer et al., 1997). Besides, only the branches with complete meristem were selected, especially for the newly formed first-order roots (i.e., pioneer roots). Because if the apex/meristem of a first-order root died or ceased to develop, and some higher-order roots would take over the functions of first-order root, then the growth and branching pattern of whole root system would change greatly (Fitter, 2002). Therefore, it was crucial to select the appreciate branches for root dissection and following analysis. Root branches with a dead first-order root apex would be excluded. And for the targeted root branches (the first-order root having alive apex), we would prefer the ones containing complete root meristem in high-order roots. Based on the above criteria, 4–8 root branches per treatment were selected for the anatomy and morphology measurement within each species. All the selected root branches were cleaned again gently, and dissected into different root developmental orders. All the individual root was labeled, then scanned with a digital scanner (Epson Expression 10000XL, Epson Telford Ltd., Suwa Nagano, Japan), scanned root images were analyzed using WinRHIZO (Pro2004b) software (Regent Instruments Company, Canada) to obtain the root diameter and length.

After that, each root was prepared for the anatomy measurement based on the procedure introduced by Guo et al. (2008a). All the roots were cut at 0–2 mm from the apex. For a better PG observation of first-order roots with larger diameter and root length, root cross sections were also prepared at every 2 mm interval from root proximal to basal (the first-order roots in Figures 2–4). Then, all root cross sections were photographed under a biological microscope (Olympus Electronics Inc., Tsukuba, Japan) equipped with a Motic 3,000 CCD camera (Motic Corporation, Xiamen, China). Root anatomy traits were measured to the nearest 1 μm using Motic Images Advanced 3.2 software, including the diameter of stele and conduit. Besides, the number of PGs and conduits were also recorded.

All the first-order roots and 30 randomly selected high-order roots were used for the morphology and anatomy measurements. Additionally, except few lost and abnormal roots, there were a total of 1,191, 445 and 205 roots used for the PG comparison analysis in *J. mandshurica*, *F. mandshurica* and *P. amurense*, respectively. In order to ensure the sample size of first-order root was enough for PG frequency analysis, another 9, 19 and 19 first-order roots were also chosen in three tree species, respectively.

### Statistical Analysis

The specific hydraulic conductivity (Ks) was calculated through Hagen–Poiseuille law (Tyree and Ewers, 1991):


$$Ks=\left(\pi \rho /128\eta Aw\right)\overset{n}{\underset{i=1}{\sum }}d_{i}^{4}$$

where Ks is theoretical axial hydraulic conductivity along a root, *ρ* is the density of water (where temperature was set at 18°C, the mean temperature in surface soil), *η* is the dynamic viscosity, *di* is the diameter of the *i*th conduit and *n* is the number of the conduits in the xylem.

A three-way ANOVA was conducted to evaluate the main effects of three factors (i.e., species, PG and treatment) and their interactions on the root morphological and anatomical traits. Considering the sample sizes and PG composition, in a given root order, we pooled the PG data from all selected root branches, then calculated the proportion of different PGs. After that, chi-square test (*p* = 0.05) was used to examine the influence of fertilization on PG composition in each root order. Besides, correlations between pairwise root traits were determined separately using Pearson’s correlation coefficient. The slopes and intercepts of correlations between treatments were compared using an analysis of covariance (ANCOVA). All statistical analyses were performed using SPSS 19.0 (IBM Corp., Armonk, NY, USA) and data visualizations were made using SigmaPlot 10.0 (Systat Software Inc., San Jose, CA, USA).

## Results

### Distribution of PG Within Fine Root System

**Figure 5:**
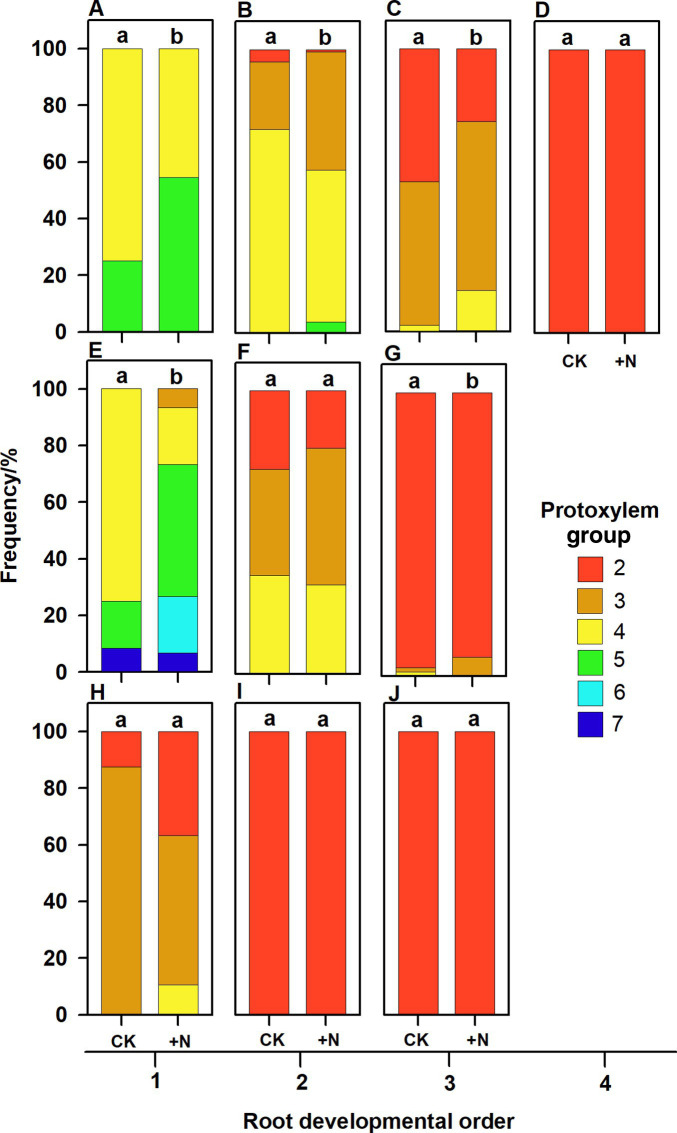
Protoxylem group composition in different root developmental order for *Juglans mandshurica* **(A–D)**, *Fraxinus mandshurica* **(E–G)** and *Phellodendron amurense* **(H–J)** under control (CK) and fertilization (+N), respectively. Different lower-case letters indicate significant differences in composition between control and fertilization within each root developmental order according to chi-square test (*p* < 0.05).

The number of PG showed large inter- and intra-specific variations (Figures 2–5; Supplementary Figure S1). Specifically, there were 2–5, 2–7 and 2–4 PGs in *J. mandshurica*, *F. mandshurica* and *P. amurense*, respectively. For the fine roots with primary growth, the number of PG did not change (specifically the proximal part of first-order root, Figures 2A,B, 3A,B, 4A–C). However, with the activity of vascular cambium, primary roots progressed to secondary growth, more and wider conduits were observed in stele, the arch nature of xylem pole lost (the basal part of first-order root, Figures 2E, 3C, 4D).

In three examined species, despite some roots of different orders had same PG number, root PG number generally decreased with ascending root developmental order (Figures 2–5). In this study, most first-order roots had four or five PGs in *J. mandshurica* and *F. mandshurica*, and three or four PGs in *P. amurense*, while nearly all the proximal and highest-order roots had only two PGs across three species (Figures 5D,G,J).

### Root Branching Architecture and Individual Root Functional Traits Related to the PG

**Figure 6:**
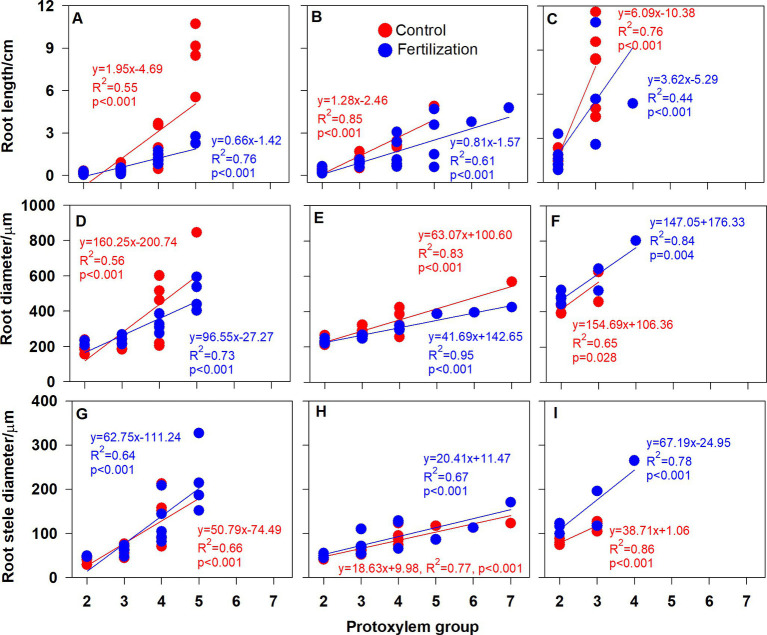
Relationship between the root protoxylem groups number and root length, root diameter and root stele diameter in *Juglans mandshurica* **(A,D,G)**, *Fraxinus mandshurica* **(B,E,H)** and *Phellodendron amurense* **(C,F,I)**, respectively.

**Figure 7:**
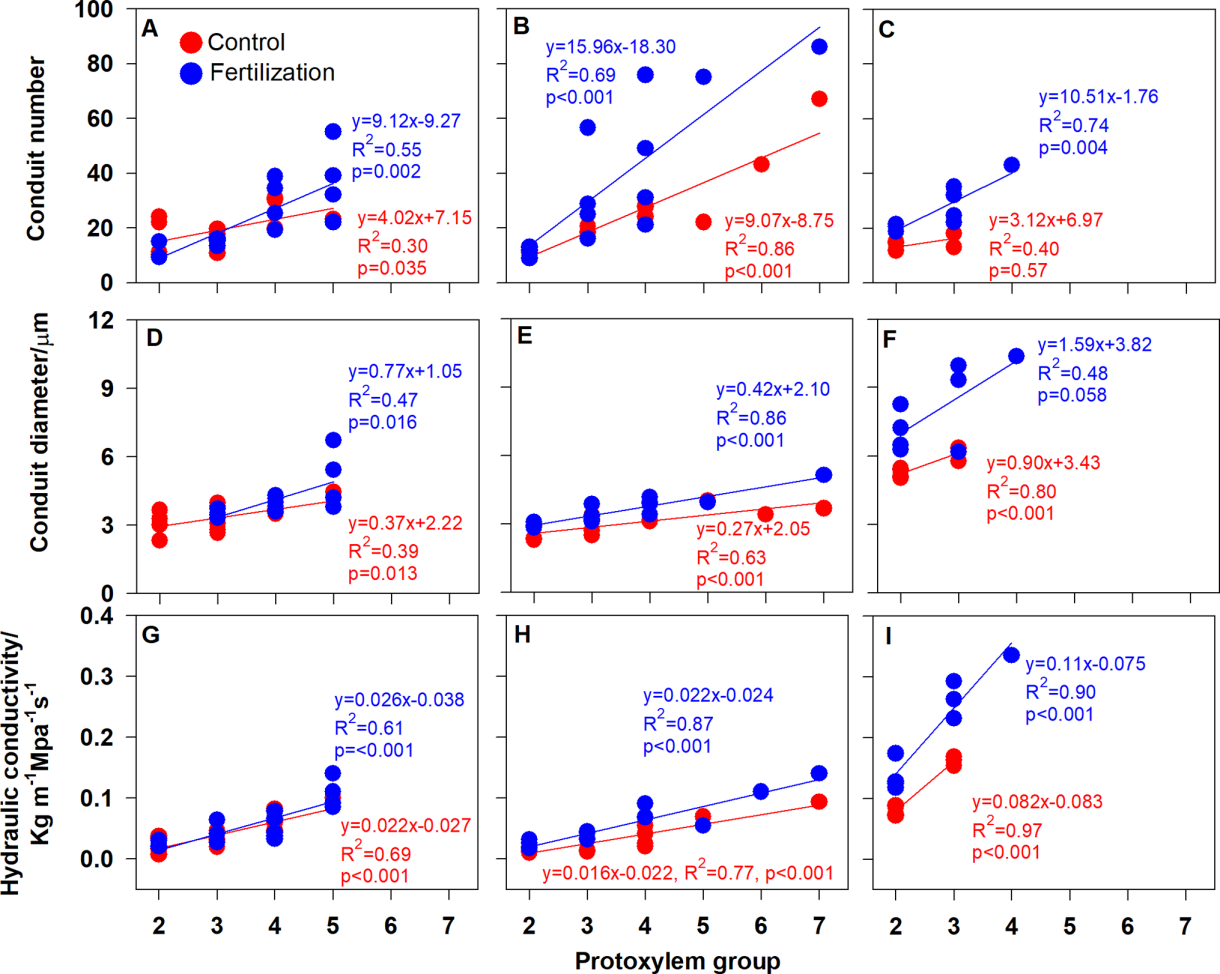
Relationship between the root protoxylem groups number and the number and diameter of root conduit, as well as hydraulic conductivity in *Juglans mandshurica* **(A,D,G)**, *Fraxinus mandshurica* **(B,E,H)** and *Phellodendron amurense* **(C,F,I)**, respectively.

Root branching architecture showed substantial interspecific variations, strongly associated with PG arrangement. The number of PG in first-order roots determined the lateral roots’ spatial distribution (Figures 2–4). More laterals emerged from parent roots with more PGs, especially for *J. mandshurica* and *F. mandshurica*, forming dichotomous root systems with thin and densely branched fine roots (Figures 2, 3). By contrast, the PG composition of parent roots was relatively simple in *P. amurense*, less laterals expanded into a herringbone architecture containing thick and sparsely branched fine roots (Figure 4). Despite these wide variations in root architecture, with the increase of PG number, significant increases were also observed in all the root length, root diameter and stele diameter (Figure 6), as well as the number and diameter of conduit and hydraulic conductivity (Figure 7), these positive correlations were independent with species and nutrient availability.

### Plasticity of Root PG, Morphology and Anatomy to Nutrient Addition

After nutrient addition, the frequency of roots with high PG increased across three tree species, significantly in first three order roots for *J. mandshurica* (Figures 5A–C; *p* < 0.05), the first three order roots for *F. mandshurica* (Figures 5E,G; *p* < 0.05). Generally, the diameters of root and conduit, as well as hydraulic conductivity increased under nutrient enrichment across species (Figures 6, 7; Table 1). Additionally, nearly all the root traits were significantly affected by the species, PG and treatment, but not their interactions (Table 1).

**Table 1:** Results of three-way (species × protoxylem group × treatment) factorial ANOVA of root morphological and anatomical traits.

	df	Root length	Root diameter	Root stele diameter	Conduit diameter	Conduit number	Hydraulic conductivity
Species	2	**<0.001**	**<0.001**	**<0.001**	**<0.001**	**0.008**	**<0.001**
Protoxylem group	6	**<0.001**	**<0.001**	**<0.001**	**<0.001**	**<0.001**	**<0.001**
Treatment	1	**<0.001**	0.515	0.647	**0.048**	0.121	**0.008**
Species × Protoxylem group	12	**<0.001**	**0.007**	**0.016**	**0.034**	0.296	**<0.001**
Species × Treatment	2	0.565	0.076	**0.012**	**<0.001**	**<0.001**	**<0.001**
Protoxylem group × Treatment	6	**<0.001**	0.321	0.071	0.799	0.597	0.002
Species × Protoxylem group × Treatment	12	0.417	0.266	**0.021**	0.939	**0.011**	0.127

Values in bold type indicate significant effects.

It is also worth noting that with the increased nutrient availability, the slopes of correlations between PG and root morphology became milder (except for the root diameter in *P. amurense*, Figures 6A–F), but steeper in the ones between PG and hydraulic traits (Figures 6G–I,7), and these changes in slopes were nearly not significant between treatments (Supplementary Table S1). However, the intercepts of correlations between PG and hydraulic parameters in *F. mandshurica* and *P. amurense* differed greatly between treatments (*p* < 0.05; Supplementary Table S1).

## Discussion

Here, we explored the arrangement of PGs and their responses to increased nutrient availability across woody species in the field. To the best of our knowledge, this is the first report to demonstrate the heterogeneity and plasticity of root PG based on the root developmental order. The PG number decreased with ascending root developmental order, and which was also tightly associated with other functional traits. These patterns were generally common in *J. mandshurica* and *F. mandshurica* with thin and densely branched root system, as well as the *P. amurense* with thick and sparsely branched root system. And the frequency of roots with high PG increased after nutrient addition across three species. Therefore, the heterogeneity and plasticity of individual roots indicated here would supply important implications for understanding how roots cope with changing environmental conditions.

### Tight Linkage Between Root PG and Functional Traits

Previous studies had exhibited great variations in root branching, morphology and anatomy across three examined species (Wang et al., 2016, 2020), herein, we further verified that PG showed strikingly large interspecific variations. Root length, diameter and stele diameter, as well as hydraulic conductivity significantly positive correlated with the PG number, supporting our first hypothesis. Similarly, some researchers also reported that PG number is strongly and broadly associated with individual root traits in woody (Wilcox, 1962; Horsely and Wilson, 1971; Zadworny and Eissenstat, 2011; Baba et al., 2018), herbaceous (Jackson, 1922; Draye et al., 1999; Mellor et al., 2019) and fern (Wardlaw, 1928) species.

Why do root functional traits positively relate to the number of PG? This might be related with the carbon allocation within root system. In a root cross section, with more PGs means more protophloems, because protoxylem elements alternate with protophloem (Esau, 1977; Parizot et al., 2008). Compared with the low PG roots, high PG roots would be enhanced in the axial transportation capacity of photosynthates (Kong et al., 2021), indicating a priority for carbon allocation (Aguirrezabal et al., 1993). Consequently, more carbohydrates would be available for root thickening and elongation, as well as the construction of vascular tissue (Figures 6G–I). In this study, increased hydraulic conductivity was associated with more and wide conduits (Figures 7A–F). Besides, high PG roots were generally pioneer roots, which extended rapidly in the soil and produced low PG fibrous roots quickly (Ding et al., 2020). Those high PG roots needed to develop transport capacity rapidly to avoid becoming a bottleneck to the absorbed water of the developing low PG roots (Bagniewska-Zadworna et al., 2012). Therefore, these significantly positive correlations between root functional traits and PG number may be universal across different plant species. Given the extensive and profound impact of root PG, which should be taken into consideration in the root classification, offering useful insights for further investigation of root traits diversity and their ecological adaptations (Freschet et al., 2021a,b).

Additionally, high PG roots were generally dense branched, especially for the first-order roots in three examined species. This might be associated with the regular distribution of lateral root primordium. At the level of cellular anatomy, only the pericycle cells adjacent to protoxylem poles are involved in lateral root initiation (Esau, 1977; Parizot et al., 2008). Therefore, more lateral root primordiums would emerge from the pericycle in high PG roots (Supplementary Figure S1), leading to more complex laterals spatial distribution (Winter, 1932; Yarbrough, 1949; Pulgarin et al., 1988) and forming a dichotomous root architecture (McMichael et al., 1987).

### Hierarchy of PG Along Root Developmental Order

Our results supported the second hypothesis that the number of PG generally decreased with ascending root order, despite there were some overlaps in some orders. Similar results were also found in *Pinus resinosa* (Fayle, 1975). This pattern might be related to the inherent hierarchy within root system (Baba et al., 2019), reflecting the differences of individual roots in carbon allocation sequence (Aguirrezabal et al., 1993), construction cost (Hishi et al., 2006) and survival strategy (Hishi and Takeda, 2005a,b). As discussed above, first-order roots were at a distinct advantage in carbon investment for tissue construction, then these high PG roots lived longer than the low PG ones (Hishi and Takeda, 2005b). Crucially, the death of first-order roots (i.e., the thick framework pioneer roots) would entail the death of all its laterals, exerting huge influence on the framework functions and resource acquisition (Guo et al., 2008b), which was not optimal for the cost–benefit of carbon investment (Eissenstat, 1992). By contrast, the death of a high-order root with low PG has less impact to the overall functionality of root system.

Although some different order roots had same PG number, their morphology and anatomy varied widely due to the diverse branching positions (our unpublished data). The strict hierarchy of PG and root functional traits along developmental order were independent of species and treatments, suggesting the intrinsic mechanism of building stable root systems. Therefore, heterogeneous individual roots would be functionally integrated by PG for a better root classification (Baba et al., 2018, 2019).

In our study, all examined root branches emerged from thicker woody roots (2–5 mm in diameter), it was convincing that these coarse woody roots also had high PGs at earlier primary development stage, although the arch nature of the xylem poles of woody roots had lost because of the activity of vascular cambium (Gambetta et al., 2013). Previous studies also reported that the number of PG did not change throughout the life cycle of individual root (Wilson and Horsley, 1970; Hishi and Takeda, 2005b). For the high PG roots, thicker diameter and larger carbon storage could enable them to defend against biotic and abiotic challenges, and those roots tended to undergo secondary growth (Hishi and Takeda, 2005b; Figure 1). In contrast, the low PG roots were at high risk of mortality and finally died as primary, especially for the thin fibrous roots (Zadworny and Eissenstat, 2011), because they were sensitive to environmental changes, such as soil freeze–thaw (Yin et al., 2017), herbivores feeding (Sun et al., 2011) and soil disturbance (Hishi and Takeda, 2005a). Therefore, we speculate that the decreased PG number with ascending root developmental order is general within the root branches having alive meristem. Additionally, although there were wide differences in PG compositions, root diameter and length among root developmental order, all these unbranched root tips would be categorized into one group based on the stream order (Pregitzer et al., 2002; Guo et al., 2008a). Consequently, it was necessary to differentiate the primary pioneer roots having high PG from finest fibrous roots with low PG in future rot researches using stream order or other method (Freschet et al., 2021a,b).

### Increased Frequency of High PG After Nutrient Addition

Our results also supported the third hypothesis that the frequency of newly formed roots with high PG increased across three species after nitrogen addition. Although the internal mechanism is still unclear, which might be related with the changes of hormones after fertilization, because auxin could cause an increase in root PG number (Aloni et al., 2006; Baba et al., 2018). More importantly, auxin content increased after root pruning (Feng et al., 2022), and exogenous nutrient addition could influence the auxin distribution, i.e., nitrate inducing auxin signaling in the pericycle (Perotti et al., 2021), ammonium affecting auxin diffusion toward the overlying cells of root vasculature (Meier et al., 2020). Therefore, more accumulation and targeted distribution of IAA occurred in the root vascular bundle, ultimately inducing more PGs in fertilized roots.

Besides, increased frequency of high PG in fertilized roots might be associated with their enhanced resource acquisition ability. With the improved accessibility of soil nutrients, root absorptive efficiency was greatly increased (Jia et al., 2011), inducing nutrient accumulation in root tissue after fertilization (Wang et al., 2018). According to the principle of functional balance between absorption-transportation (Kong et al., 2017), individual roots would optimize their structures to maintain the functional equilibrium when their absorptive ability was enhanced, in term of more PGs increasing the root axial transportation capacity of absorbed resources (Figure 5).

Although the increased frequency of high PG roots after fertilization was found across three species, which was only significant in *J. mandshurica* and *F. mandshurica,* but not in *P. amurense*. Coupled with the simple composition of PG in *P. amurense*, we speculate this might be associated with the plant phylogeny. *P. amurense* was a thick-root species of basal clades, while the other two species were thin-root species of recently diverged clades (Gu et al., 2014). Previous studies had confirmed that ancestral thick-roots species were relatively conservative in root construction (Xia et al., 2021) and responding to environmental changes (Eissenstat et al., 2015; Liu et al., 2015). Therefore, simple composition and poor plasticity of root PG seemed to be other manifestations of phylogenetic conservatism of thick-root species. Therefore, future studies sampling more species spanning wide evolutionary scales are urgently needed for a better understanding the relationship between plant phylogeny and root PG arrangement (Freschet et al., 2021a).

## Conclusion

Root protoxylem groups are strongly associated with individual root functional traits. Specifically, the number of PG showed significantly positive relations to root length, diameter and stele, as well as hydraulic conductivity. More importantly, such linkages were still existent under nutrient addition. The PG number decreased with the increase of root developmental order, i.e., proximal first-order roots had more PGs than the distal high-order ones. Furthermore, the frequency of high PG roots generally increased after nutrient addition. Therefore, the arrangement of PG was affected by both internal and external factors. These findings highlight the hierarchy, heterogeneity and plasticity of PG within root branches, all of them are crucial for the understanding the key roles of different individual roots in the root system construction.

## Data Availability Statement

The original contributions presented in the study are included in the article/Supplementary Material, further inquiries can be directed to the corresponding author.

## Author Contributions

ZL, YW, and JG designed the experiments. ZL and YW conducted yield experiments, performed data analysis, and wrote the paper. ZL, SW, WW, and YW processed laboratory work. All authors have read and approved the manuscript.

## Funding

This research was jointly supported by the National Natural Science Foundation of China (32101514 and 31870608).

## Conflict of Interest

The authors declare that the research was conducted in the absence of any commercial or financial relationships that could be construed as a potential conflict of interest.

## Publisher’s Note

All claims expressed in this article are solely those of the authors and do not necessarily represent those of their affiliated organizations, or those of the publisher, the editors and the reviewers. Any product that may be evaluated in this article, or claim that may be made by its manufacturer, is not guaranteed or endorsed by the publisher.

## Supplementary Material

Supplementary Figure S1 Typical fine roots with different root protoxylem groups and lateral primordium emerging from pericycle cell adjacent to the protoxylem poles in three hardwood species.
