# Corrigendum: The hierarchy of protoxylem groupings in primary root and their plasticity to nitrogen addition in three tree species

**DOI:** 10.3389/fpls.2023.1291059

**Published:** 2023-10-16

**Authors:** Zhongyue Li, Siyuan Wang, Wenna Wang, Jiacun Gu, Yan Wang

**Affiliations:** ^1^Mountain Tai Forest Ecosystem Research Station of State Forestry and Grassland Administration, College of Forestry, Shandong Agricultural University, Tai’an, China; ^2^Key Laboratory of Sustainable Forest Ecosystem Management-Ministry of Education, School of Forestry, Northeast Forestry University, Harbin, China; ^3^Institute of Tropical Agriculture and Forestry, Hainan University, Haikou, China

**Keywords:** hardwood species, fine root, root developmental order, protoxylem group, morphology, anatomy, fertilization

In the published article, there were errors in **Figures 5**, **6** and **7** as published. The word “proxylem” was misspelled. This should have been written as “protoxylem “. The corrected **Figures 5**, **6** and **7** and their captions “FIGURE 5. Protoxylem group composition in different root developmental order for *Juglans mandshurica* (A-D), *Fraxinus mandshurica* (E-G) and *Phellodendron amurense* (H-J) under control (CK) and fertilization (+N), respectively. Different lower-case letters indicate significant differences in composition between control and fertilization within each root developmental order according to chi-square test (P<0.05)”, “FIGURE 6. Relationship between the root protoxylem groups number and root length, root diameter and root stele diameter in *Juglans mandshurica* (A, D, G), *Fraxinus mandshurica* (B, E, H) and *Phellodendron amurense* (C, F, I), respectively” and “FIGURE 7. Relationship between the root protoxylem groups number and the number and diameter of root conduit, as well as hydraulic conductivity in *Juglans mandshurica* (A, D, G), *Fraxinus mandshurica* (B, E, H) and *Phellodendron amurense* (C, F, I), respectively” appear below.

**Figure 5:**
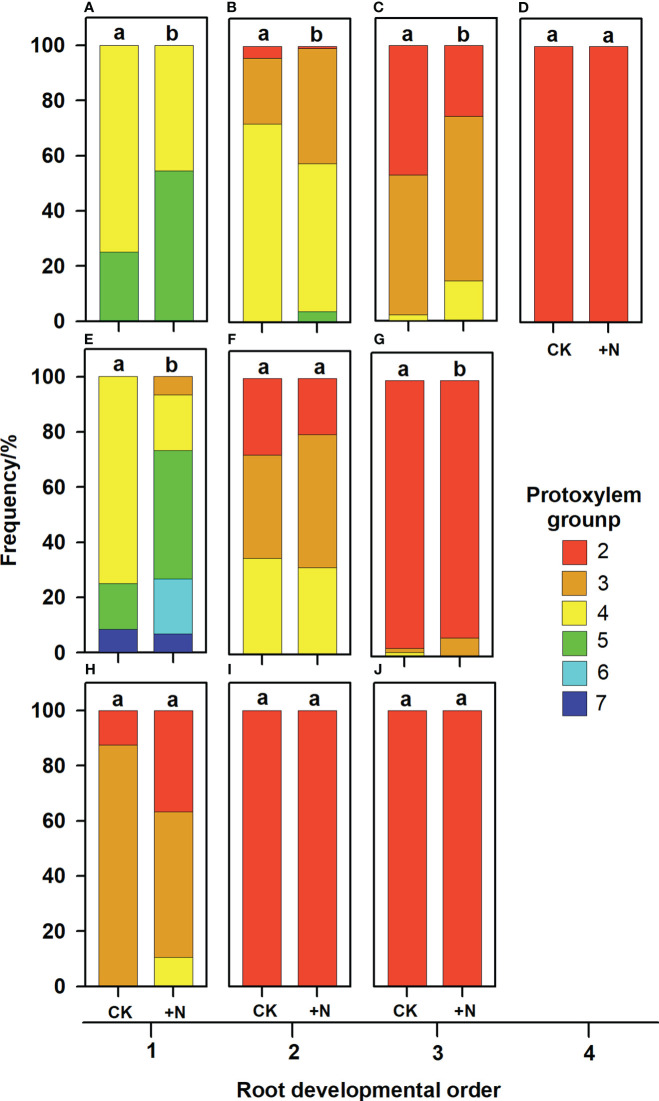
Protoxylem group composition in different root developmental order for *Juglans mandshurica* **(A-D)**, *Fraxinus mandshurica* **(E-G)** and *Phellodendron amurense* **(H-J)** under control (CK) and fertilization (+N), respectively. Different lower-case letters indicate significant differences in composition between control and fertilization within each root developmental order according to chi-square test (*P*<0.05).

**Figure 6:**
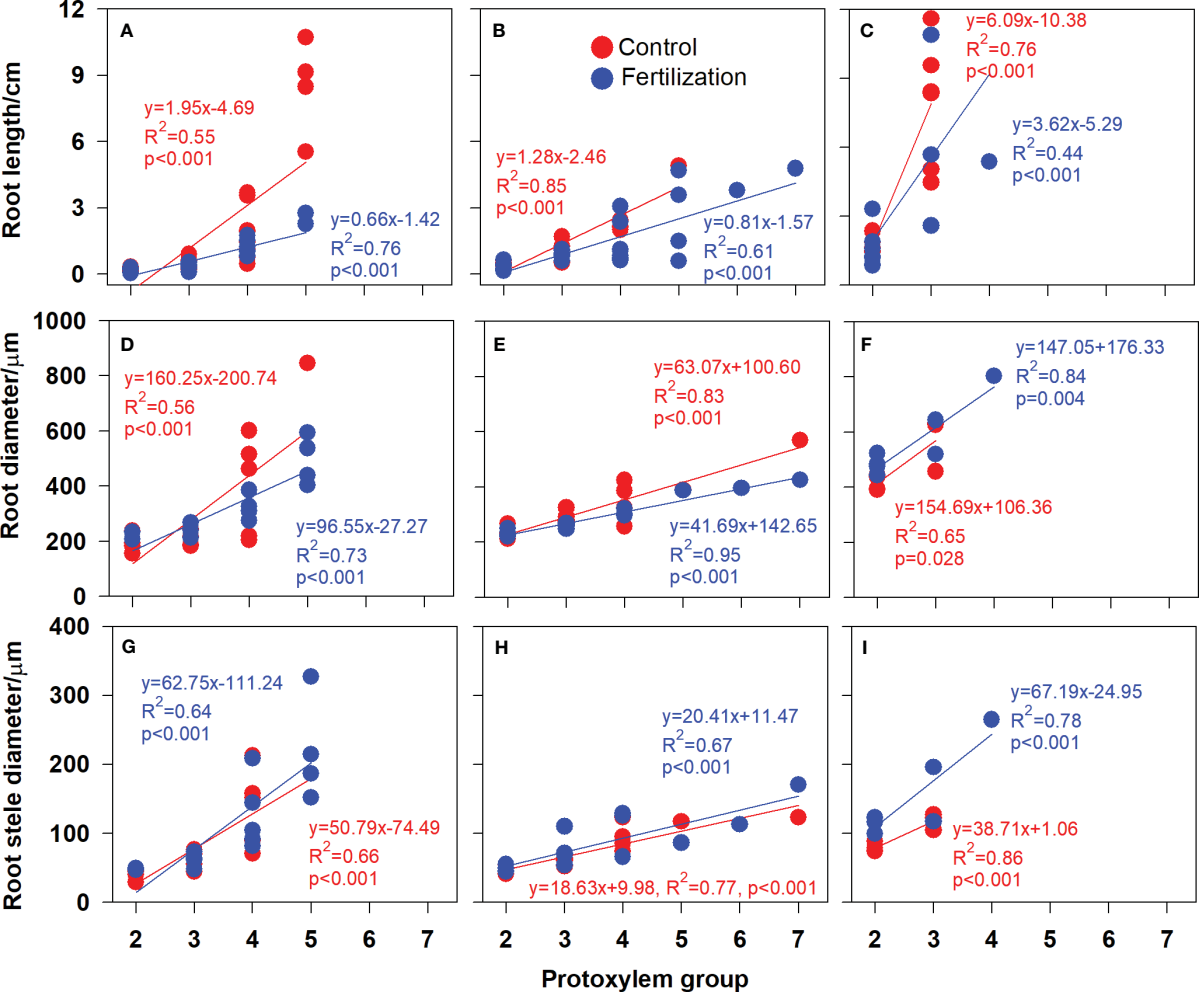
Relationship between the root protoxylem groups number and root length, root diameter and root stele diameter in *Juglans mandshurica* **(A, D, G)**, *Fraxinus mandshurica* **(B, E, H)** and *Phellodendron amurense* **(C, F, I)**, respectively.

**Figure 7:**
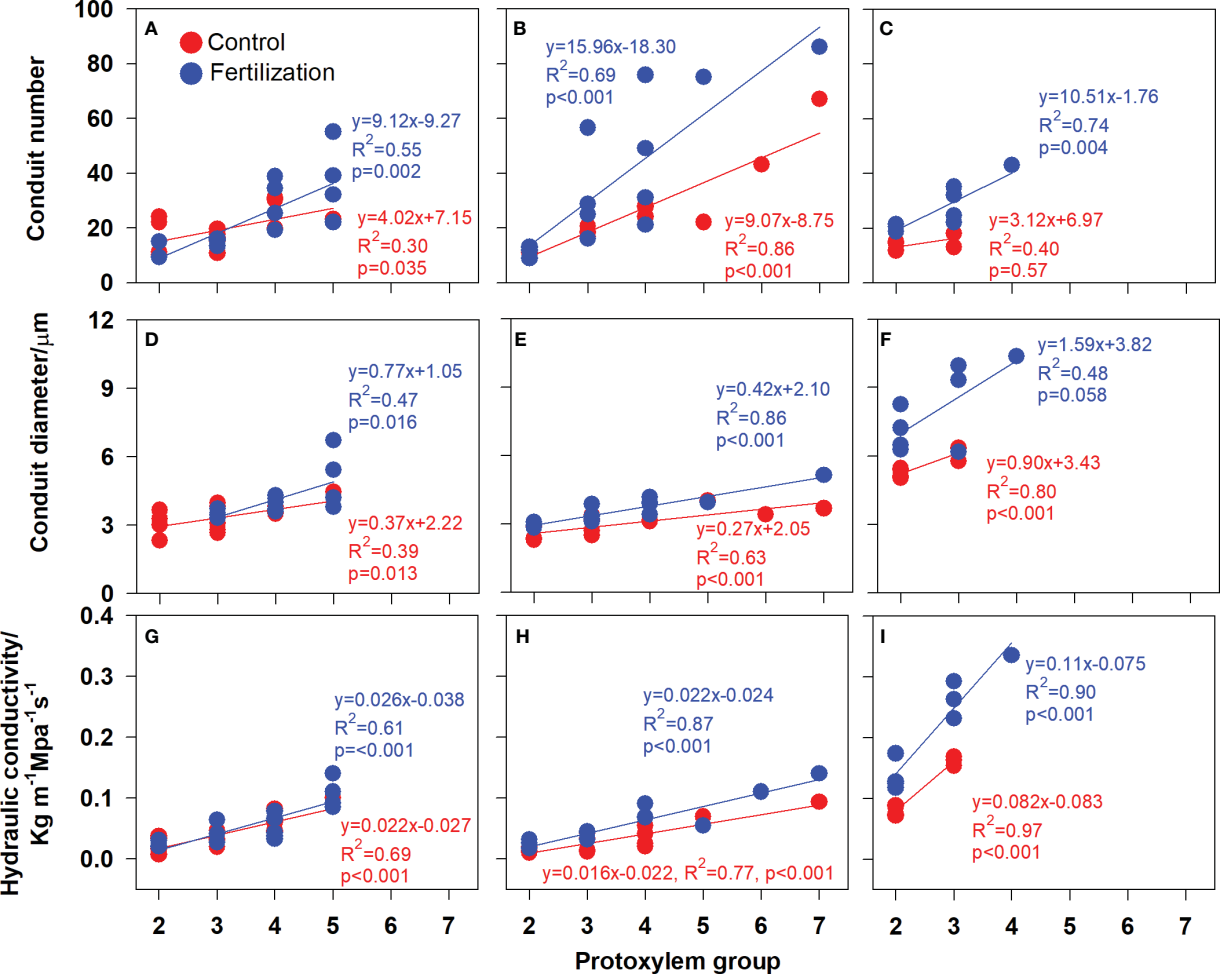
Relationship between the root protoxylem groups number and the number and diameter of root conduit, as well as hydraulic conductivity in *Juglans mandshurica* **(A, D, G)**, *Fraxinus mandshurica* **(B, E, H)** and *Phellodendron amurense* **(C, F, I)**, respectively.

The authors apologize for this error and state that this does not change the scientific conclusions of the article in any way. The original article has been updated.

