# Supplementary figures and images for: The Hierarchy of Protoxylem Groupings in Primary Root and Their Plasticity to Nitrogen Addition in Three Tree Species
Source: Front Plant Sci. 2022 Jun 23;13:903318. doi: 10.3389/fpls.2022.903318 (PMC9260270; doi:10.3389/fpls.2022.903318)

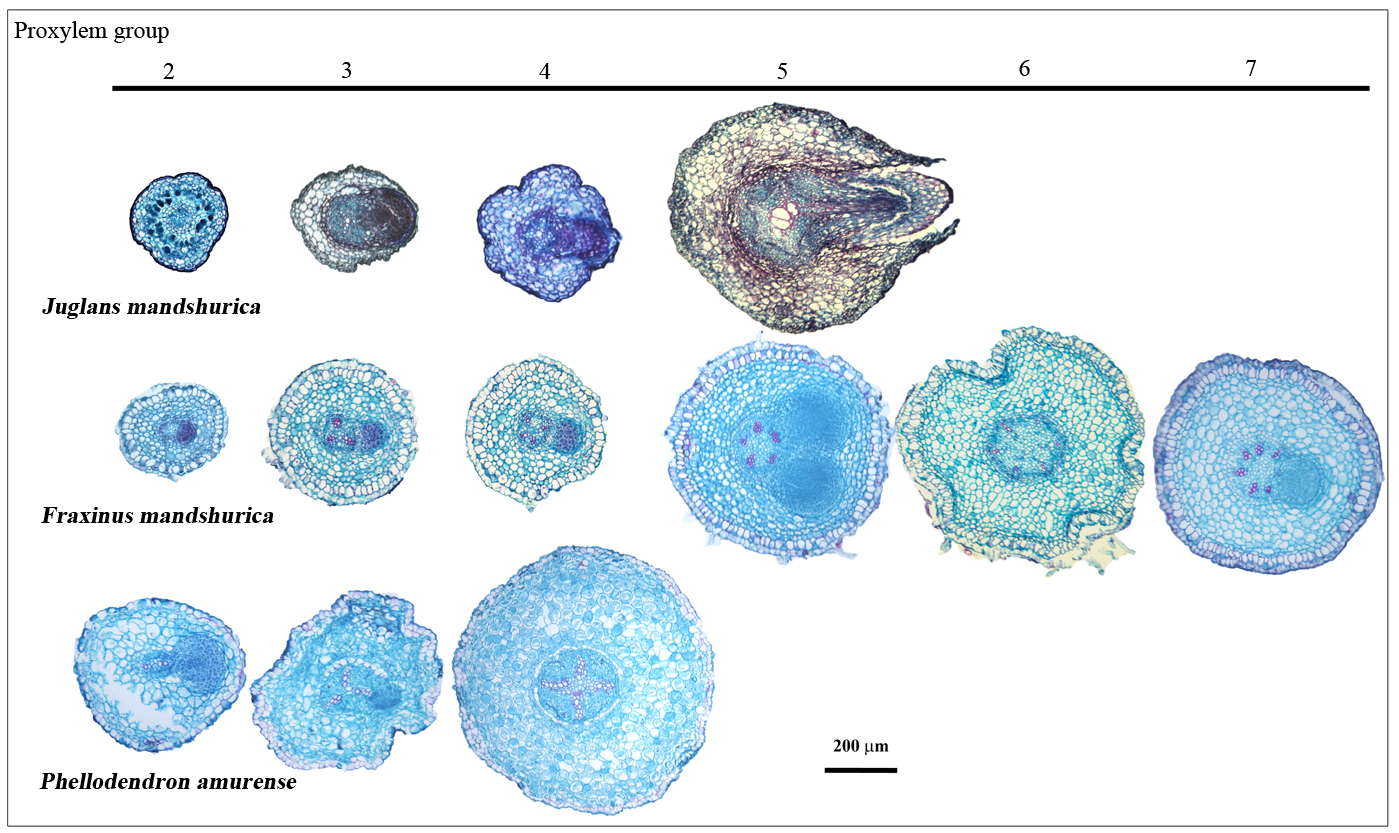

Supplement: Supplementary Figure S1 — Typical fine roots with different root protoxylem groups and lateral primordium emerging from pericycle cell adjacent to the protoxylem poles in three hardwood species. [file Image_1.TIF]
