# Supplementary material for: The Hierarchy of Protoxylem Groupings in Primary Root and Their Plasticity to Nitrogen Addition in Three Tree Species
Source: Front Plant Sci. 2022 Jun 23;13:903318. doi: 10.3389/fpls.2022.903318 (PMC9260270; doi:10.3389/fpls.2022.903318)
Supplement: Supplementary file 2 [file Table_1.DOCX]

**Table S1**. The results of ANCOVA of slope and intercept of the liner regression between PGs and root functional traits
